# Endocannabinoid Regulation of Acute and Protracted Nicotine Withdrawal: Effect of FAAH Inhibition

**DOI:** 10.1371/journal.pone.0028142

**Published:** 2011-11-30

**Authors:** Andrea Cippitelli, Giuseppe Astarita, Andrea Duranti, Giovanni Caprioli, Massimo Ubaldi, Serena Stopponi, Marsida Kallupi, Gianni Sagratini, Fernando Rodrìguez de Fonseca, Daniele Piomelli, Roberto Ciccocioppo

**Affiliations:** 1 School of Pharmacy, Pharmacology Unit, University of Camerino, Camerino, Italy; 2 Department of Pharmacology, University of California Irvine, Irvine, California, United States of America; 3 Department of Biomolecular Sciences, Medicinal Chemistry and Technology Unit, University of Urbino “Carlo Bo”, Urbino, Italy; 4 School of Pharmacy, Medicinal Chemistry Unit, University of Camerino, Camerino, Italy; 5 Fundación IMABIS, Hospital Carlos Haya de Málaga, Malaga, Spain; 6 Drug Discovery and Development, Italian Institute of Technology, Genova, Italy; Sapienza University of Rome, Italy

## Abstract

Evidence shows that the endocannabinoid system modulates the addictive properties of nicotine. In the present study, we hypothesized that spontaneous withdrawal resulting from removal of chronically implanted transdermal nicotine patches is regulated by the endocannabinoid system. A 7-day nicotine dependence procedure (5.2 mg/rat/day) elicited occurrence of reliable nicotine abstinence symptoms in Wistar rats. Somatic and affective withdrawal signs were observed at 16 and 34 hours following removal of nicotine patches, respectively. Further behavioral manifestations including decrease in locomotor activity and increased weight gain also occurred during withdrawal. Expression of spontaneous nicotine withdrawal was accompanied by fluctuation in levels of the endocannabinoid anandamide (AEA) in several brain structures including the amygdala, the hippocampus, the hypothalamus and the prefrontal cortex. Conversely, levels of 2-arachidonoyl-*sn*-glycerol were not significantly altered. Pharmacological inhibition of fatty acid amide hydrolase (FAAH), the enzyme responsible for the intracellular degradation of AEA, by URB597 (0.1 and 0.3 mg/kg, i.p.), reduced withdrawal-induced anxiety as assessed by the elevated plus maze test and the shock-probe defensive burying paradigm, but did not prevent the occurrence of somatic signs. Together, the results indicate that pharmacological strategies aimed at enhancing endocannabinoid signaling may offer therapeutic advantages to treat the negative affective state produced by nicotine withdrawal, which is critical for the maintenance of tobacco use.

## Introduction

Nicotine is one of the most widely used addictive drugs, and smoking tobacco is the most common form of substance abuse. The World Health Organization (WHO) estimates that there are 1.25 billion smokers worldwide, representing one third of the global population over the age of 15. Further estimates show that 5 million deaths occur each year as a direct result of tobacco use, making nicotine abuse the largest single preventable cause of death worldwide [1].

Nicotine, same as other abused drugs, has reinforcing psychoactive effects that lead to its self-administration. Although positive reinforcement of nicotine is less effective than other abused drugs [2], smoking cessation leads to an aversive state that serves as a negative reinforcer to the maintenance of the tobacco consumption in smokers [3], [4], [5]. Clinical studies have shown that tobacco cessation leads to expression of withdrawal symptoms such as irritability, anxiety, depressed mood, increased hunger, restlessness, difficulty concentrating, sleep disturbances, weight gain, decreased heart rate and craving for tobacco [6], [7], [8]. Emergence of negative withdrawal symptoms represent a primary reason for the persistence of smoking and represent one of the major obstacles for successful detoxification.

To shed light on nicotine withdrawal mechanisms several animal models have been developed using rats [9], [10], [11], [12], [13] and mice [14], [15], [16], [17], [18]. The rodent nicotine abstinence syndrome is characterized by somatic manifestations and is accompanied by aversive motivational and affective states. Such symptoms can occur spontaneously after nicotine exposure cessation [11], [19] or can be precipitated by nicotinic acetylcholine receptor antagonists [12],[20] and reversed by further nicotine exposure [11]. This evidence suggests that nicotinic receptors play a key role in the neurobiology of nicotine withdrawal. However, nicotine affects the function of several neurotransmitter systems, including dopamine, opioid peptides, serotonin and glutamate systems [5], [21]. Furthermore, functional interactions between nicotine and the endocannabinoid systems in relation to addiction-related processes have been described. Particularly, an overlapping distribution of cannabinoid and nicotinic acetylcholine receptors was reported in several brain areas including the hippocampus and the amygdala [22]. Also, cannabinoid receptor activation has been shown to modulate the release and turnover of acetylcholine in various brain regions [23], [24], [25]. In addition, converging experimental evidence indicates that these two systems facilitate each other's pharmacological and rewarding effects. For example, the co-administration of subthreshold doses of delta-9-tetrahydrocannabinol (Δ^9^- THC), the main psychoactive ingredient in *Cannabis*, and nicotine produced a significant conditioned place preference, whereas nicotine rewarding effects were absent in cannabinoid CB_1_ receptors knockout mice [26], [27]. Consistently, the CB_1_ receptor antagonist rimonabant was shown to decrease nicotine self-administration and conditioned place preference in rats [28], [29], [30]. It was also shown that acute Δ^9^-THC administration significantly decreased the incidence of mecamylamine and naloxone-precipitated nicotine withdrawal signs and ameliorated the aversive motivational consequences of nicotine withdrawal in mice [15], [31].

Together these findings raise the question to whether expression of nicotine withdrawal is associated with modifications in endocannabinoid neurotransmission. Moreover, based on these data, it would be relevant to evaluate whether pharmacological manipulations of the endogenous cannabinoid system would affect behaviors associated with the expression of nicotine abstinence syndrome. To accomplish this objective, we generated a rat model of spontaneous nicotine withdrawal following the removal of chronically implanted transdermal nicotine patches and analyzed the occurrence of ongoing somatic and affective (anxiety) withdrawal signs. Next we evaluated tissue levels of the main endocannabinoids, anandamide (arachidonoyl ethanol amide, AEA) and 2-arachidonoyl-*sn*-glycerol (2-AG) during acute (16 hours) and protracted (34 hours) nicotine withdrawal. The results show that major changes occurred for AEA, while 2-AG was minimally affected. Therefore, we used URB597, a potent and systemically active inhibitor of fatty acid amido hydrolase (FAAH), the enzyme responsible for AEA deactivation within cells [32], [33], [34], to evaluate whether an increased AEA tone modifies the expression of nicotine withdrawal.

## Materials and Methods

### Ethics Statement

All experimental procedures were conducted in adherence to the European Community Council Directive (86/609/CEE) and the National Institutes of Health Guidelines for Care and Use of Laboratory Animals. Authorization concerning the use of laboratory animals and approval of experimental procedures was provided by the Ministry of Health (Italy, dècret 85/2007-A, 1 October, 2007).

### Animals

Male Wistar rats (Charles River, Calco, Italy) weighting 275–325 g at the beginning of the experiment were used. Animals were housed in groups of two and kept on a reverse 12-h light/dark cycle (lights on 20:00–08:00 h). Each experiment was conducted with independent groups of rats during the dark phase of the cycle. The following experimental groups were used: one batch of animals (*N* = 12; 6 nicotine-exposed and 6 non-exposed controls) was used in Experiment 1 to evaluate the expression of somatic and affective nicotine withdrawal signs at 16 and 34 hours from patch removal, respectively. Another batch (*N* = 27, Experiment 2) was divided into two groups of 13 (8 nicotine and 5 vehicle) and 14 rats (8 nicotine and 6 vehicle) and was used to evaluate extracellular concentration of AEA and 2-AG at 16 and 34 hours following nicotine patches exposure. A third cohort of rats (*N* = 32, 8 nicotine naïve vehicle treated; 8 nicotine-exposed vehicle treated, 8 nicotine-exposed receiving URB597 0.1 mg/kg and 8 nicotine-exposed receiving URB597 0.3 mg/kg) was used in Experiment 3 to evaluate the effects of FAAH inhibition on the expression of somatic signs of withdrawal. Further animals (*N* = 43, 10–11 per group, divided as in the Experiment 3) served to study the effects of URB 597 on withdrawal-induced anxiety at 34 hours, as assessed in the elevated plus-maze (EPM, Experiment 4). In addition, other 27 (*N* = 9/group) nicotine naïve rats were used to test the anxiolytic effect of URB597 under basal condition. Finally, others (*N* = 31, 7–8 per group) were used in Experiment 5 to test at 34 hours the effects of the URB597 on anxiety-like behavior assessed by the shock-probe defensive burying paradigm. Additional Wistar rats (*N* = 5) were used to determine the time course of blood nicotine and cotinine levels during the dependence induction procedure.

### Drugs

Nicotine patches (NIQUITIN CQ Step 1, 21 mg/day, Glaxo, Verona, Italy) were used for dependence induction. URB597 was synthesized as previously described [35]. It was suspended in 5% PEG400, 5%TWEEN-80 and 90% saline. The compound was administered intraperitoneally (i.p.) at a volume of 1 ml/kg. URB597 (0.1, 0.3 mg/kg) or vehicle was given 120 min prior the signs' observation and locomotor performance, since FAAH inhibition by URB597 occurs rapidly (<15 minutes) and persists more than six hours [34], [36]. A 60 min pre-treatment time was used prior to elevated plus-maze or shock-probe defensive burying tests.

### Dependence induction and withdrawal

Nicotine exposure - Rats were thoroughly shaved on the back, depilated with a depilatory lotion and cleansed with water as previously described [13]. Patches were divided into 4 equal parts so that 5.2 mg/rat/day of nicotine was administered by patch applied to the shaved region. Comparable doses were previously shown to produce sufficiently high blood nicotine and cotinine levels [37] to elicit occurrence of reliable nicotine abstinence symptoms. Pieces of flexible fabric Band-Aid and waterproof tape were used to wrap the nicotine patch to improve its adherence to the rat's back. Control rats were shaved and depilated, but only the Band-Aid and waterproof tape were placed on their backs. This application procedure was repeated for 7 consecutive days. On day 8, transdermal patches were removed to study nicotine withdrawal. Such experimental procedure was repeated throughout all experiments.

Nicotine and cotinine blood level assessment - To determine levels of nicotine and cotinine in the blood, rats were sacrificed at the middle (4 days) and at the end (7 days) of the nicotine patch application procedure. Two ml of trunk blood was collected and then centrifuged at 4200 rpm at 17°C for 8 min. Following collection of the supernatant quantitative analysis of samples was determined by high performance liquid chromatography (HPLC) coupled mass spectrometry (LC-MS). All samples were assessed in triplicate. Nicotine and cotinine data are expressed as concentrations in micrograms/liter (µg/l).

Assessment of somatic withdrawal signs - According with previous studies showing that overt spontaneous nicotine withdrawal signs appear 16 h after termination of nicotine exposure and return toward baseline at 40 h [11], [12], in the current experiments we monitored physical abstinence signs 16 hours after the removal of nicotine patches. The assessment of somatic signs was performed by blind observation across 10 min. Observers counted the frequency of signs on a standard checklist of nicotine abstinence signs, as previously described [11]. The most frequently observed categories included teeth-chattering/chews, writhes/gasps, wet dog shakes/tremors and yawns. Teeth chattering were counted no more frequently than once per ten seconds.

Locomotor activity in the open field - At the same time as the assessment of somatic withdrawal signs, locomotor activity was recorded over a period of 10 min. Each rat was monitored for locomotor activity while located in the open arena (MedAssociates, St. Albans, VT). Interruptions of 10 equally spaced infrared light beams recorded total distance traveled, immobility, vertical movements, number of entries onto central zone of the arena and time spent in this zone. These last two parameters were also considered as a measure of anxiety. Activity was examined under non-familiar dim-light conditions.

Weight - Changes in body weight are thought to measure withdrawal intensity [38]. Animals were weighed each day when transdermal patches were replaced. Additionally, body weights were measured at the end of the exposure (T = 0), at 16 (T = 16) and 34 (T = 34) hours following patch removal.

#### Elevated Plus-Maze

Anxiety-like behavior was monitored using a standard elevated plus-maze apparatus as previously described [36], [39]. The time spent in open arms and entries onto open arms were used as measures of anxiety-like behavior. The EPM test was carried out 34 h following removal of nicotine patches. This time point corresponding to the late withdrawal period [11] was chosen also based on the result of Experiment 1 showing increased anxiety in the nicotine-dependent group at this time point.

#### Shock-probe defensive burying paradigm

The shock-probe defensive burying test [40], [41] was used as an additional measure for the assessment of anxiety-like behavior. The defensive burying apparatus was a modified home cage with 4 cm wood chip bedding material evenly distributed throughout the cage. One end of the cage contained a 0.75-cm hole through which a probe delivering 1.5 mA electric shock upon contact was inserted into the cage. The probe remained on throughout the test. Contacts to probe resulted in the rat piling bedding material with treading-like movements of the forepaws and shoveling movements of the head, often directed toward the shock-probe. The latency to start burying and duration of burying were used as a measure of anxiety. The test was carried out 34 h following nicotine patch removal under low light conditions. Each animal was used only once.

### Endocannabinoid levels in the brain

After 16 and 34 hours from patch removal, brains from animals previously receiving nicotine and their respective controls were removed and quickly frozen at −80°C until use. Brains were dissected and tissue levels of AEA and 2-AG were measured in discrete brain areas.

Chemical syntheses - Heptadecenoylethanolamide (HEA) was prepared by the reaction of the heptadecenoyl chloride (Nu-Chek Prep, Elysian, MN) with a 10-fold molar excess of ethanolamine (Sigma-Aldrich, St. Luis, Missouri, USA). Reactions were conducted in dichloromethane at 0–4°C for 15 min, with stirring. The product was washed with water, dehydrated over sodium sulfate, filtered, and dried under N_2_. It was identified by liquid chromatography/mass spectrometry (LC/MS) and ^1^H nuclear magnetic resonance spectroscopy. Purity was >98% by LC/MS.

Endocannabinoid extraction and analysis - Selected brain regions were punched from the frozen brains using a cryo-cut and cylindrical brain punchers (Fine Science tools, Foster City, CA, USA), as previously described [42]. The location and length of the punches were chosen based on a stereotaxic atlas [43]. Frozen punches were homogenized in 0.3 ml of methanol containing HEA and 2-heptadecanoyl-*sn*-glycerol (Nu-Chek Prep, Elysian, MN) as internal standards. Lipids were extracted with chloroform (2 vol) and washed with water (1 vol). Protein concentrations were measured using the BCA protein assay (Pierce, Rockford, IL). Organic phases were collected and dried under N_2_. Endocannabinoids were fractionated as described previously [44]. Briefly, the lipids were reconstituted in chloroform and loaded onto small glass columns packed with Silica Gel G (60-Å 230–400 Mesh ASTM; Whatman, Clifton, NJ). Endocannabinoids were eluted with 9∶1 chloroform/methanol (vol/vol). Eluates were dried under N_2_ and reconstituted in 0.1 ml of chloroform/methanol (1∶4, vol/vol) for LC/MS analyses.

LC/MS analyses - We used an 1100-LC system coupled to a 1946D-MS detector (Agilent Technologies, Inc., Palo Alto, CA) equipped with an electrospray ionization interface. Endocannabinoids were separated using a XDB Eclipse C18 column (50×4.6 mm i.d., 1.8 µm, Zorbax), eluted with a gradient of methanol in water (from 75% to 85% in 2.5 min and then to 90% in 7.5 min) at a flow rate of 1.0 ml/min. Column temperature was kept at 40°C. MS detection was in the positive ionization mode, capillary voltage was set at 3 kV and fragmentor voltage was varied from 120 V. N_2_ was used as drying gas at a flow rate of 13 liters/min and a temperature of 350°C. Nebulizer pressure was set at 60 PSI. Quantifications were performed by monitoring in selected ion monitoring mode the sodium adducts of the molecular ions (AEA, *m/z* 370.3; 2-AG, *m/z* 401.3; HEA, *m/z* 334.3; 2-HG, m/z 367.3).

### Statistics

Endocannabinoid levels were analyzed by one-way ANOVA where treatment (nicotine/controls) and time (16 h/34 h) were treated as independent factors. Different brain regions were analyzed separately. All behavioral experiments were analyzed using one-way ANOVA except for body weight analysis during dependence induction and withdrawal, which were performed by two-way ANOVA where the within subjects variable was “time” (T = 0, T = 16, T = 34 hours), and the between subjects variable was “group” (Control, Nicotine). When appropriate, *post hoc* comparisons were carried out by Newman-Keuls test.

## Results

### Blood nicotine and cotinine levels

Application of the nicotine patch resulted in elevated levels of nicotine and cotinine in the blood.

Average nicotine levels at the middle (day 4) and at the end (day 7) of the dependence induction procedure were 691.0±11.5 and 748.3±134.3 µg/l, respectively. Levels of cotinine, the primary metabolite of nicotine, appeared to increase from 1210±174 µg/l of day 4 to 1547±109 µg/l of day 7, thus suggesting accumulation of the metabolite in the blood. The relative standard deviations resulting from the analysis in triplicate ranged from 1.3 to 2.2% for run-to-run precision, and from 3.1 to 4.4% for day-to-day precision. Blood nicotine and cotinine levels of nicotine naïve animals were undetectable.

### Experiment 1: Removal of chronically implanted transdermal nicotine patches produces spontaneous withdrawal

**Figure 1:**
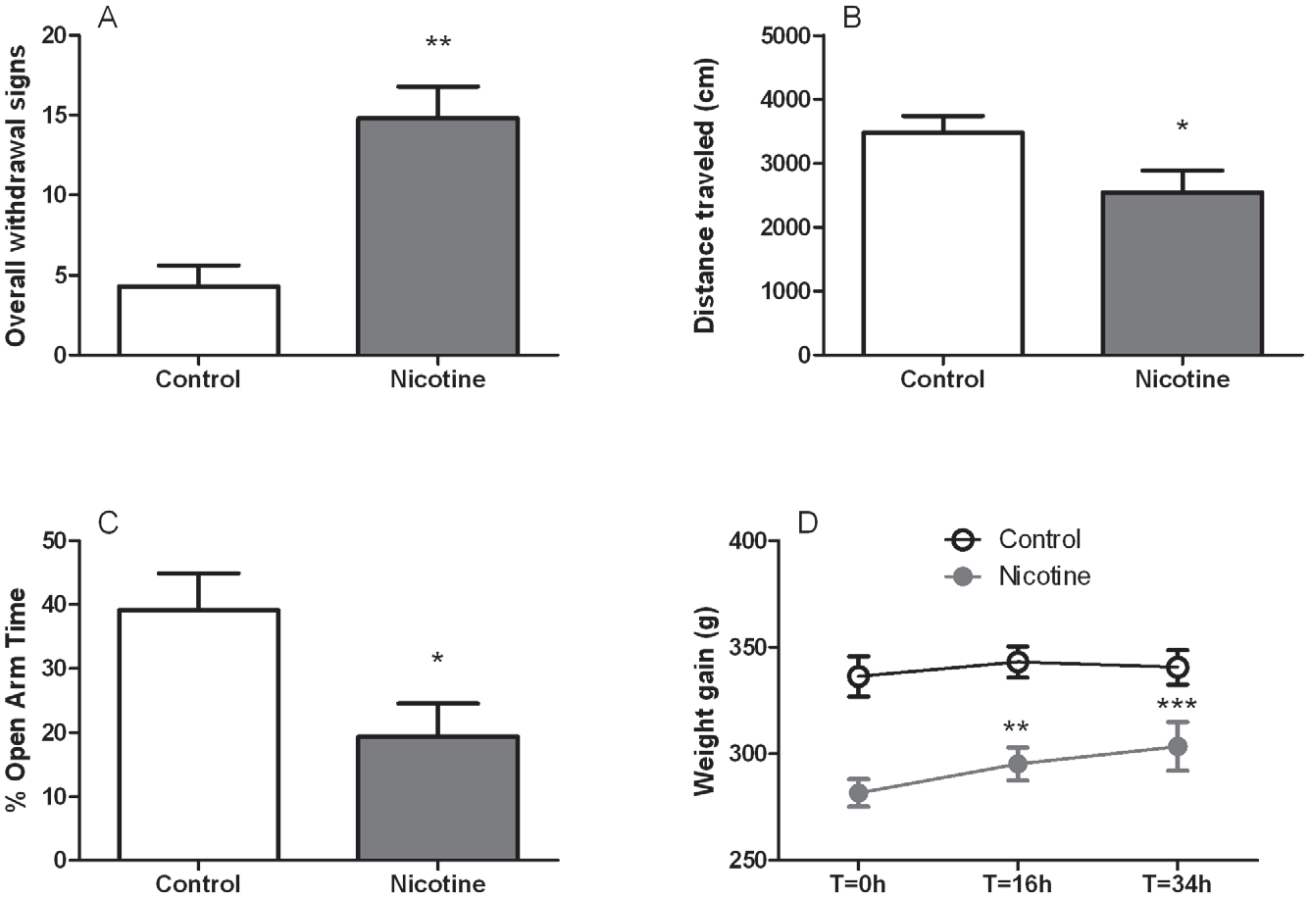
Spontaneous nicotine withdrawal measured after 7 days of nicotine exposure (5.2 mg/rat/day). Rats previously exposed to nicotine showed (**A**) increased occurrence of overall withdrawal signs compared to non-exposed controls. Overall withdrawal signs were obtained for each animal by accumulating the number of events belonging to different categories of somatic withdrawal manifestations (wet dog shakes/tremors, gasp/writhes, chattering/chews) scored at 16 hours from nicotine discontinuation. Nicotine exposed rats also showed: (**B**) decreased distance (cm) traveled in the open field at the 16-hour time point; (**C**) reduced percentage (%) in open arms time measured at 34 hours from nicotine patch removal, and (**D**) rapid body weight gain (g). Values represent the mean (±SEM) of N = 6 subjects per group. In A, B and C, *p<0.05, **p<0.01 difference vs. control. In D, **p<0.01, ***p<0.001 difference vs. nicotine group at T = 0 h.

This experiment was performed to examine whether the removal of chronically implanted transdermal nicotine patches is able to cause somatic and affective symptoms of withdrawal. The nicotine dependence induction procedure spontaneously produced a substantial increase of overall somatic withdrawal signs compared to controls ([*F*_(1,10)_ = 19.2, *p*<0.01], Figure 1A). In particular, rats receiving nicotine differed significantly from control rats in all the examined dependent variables including wet dog shakes/tremors [*F*_(1,10)_ = 20.9, *p*<0.01], teeth-chattering/chews [*F*_(1,10)_ = 5.2, *p*<0.05] and gasps/writhes ([*F*_(1,10)_ = 16.2, *p*<0.01], Table S1).

The occurrence of somatic withdrawal signs was accompanied by secondary abstinence symptoms such as decreased locomotion as reflected by reduction in the total distance travelled ([*F*_(1,10)_ = 4.7, *p* = 0.05], Figure 1B) and increased immobility time [*F*_(1,10)_ = 6.1, *p*<0.05]. Vertical locomotor activity was not affected (rearings: [*F*_(1,10)_ = 0.0, NS], Table S2).

In the same groups of rats, anxiety-like behavior was then evaluated in the EPM 34 h following patch removal. As shown in Figure 1C, rats previously treated with nicotine spent significantly less time exploring the open arms of the maze (percent open-arm time, [*F*_(1,10)_ = 6.6, *p*<0.05]). Percentage of open-arm entries resulted as a less reliable measure of anxiety-like behavior [*F*_(1,10)_ = 0.8, NS]. Also, the number of closed-arm entries was not changed at this time point ([*F*_(1,10)_ = 0.4, NS], Table S3).

Additional evidence for the occurrence of withdrawal following chronic application of nicotine patches was provided by increased weight gain during withdrawal. The dependence induction procedure caused a progressive drop in body weight from day 1 (334.5±9.4 g) to day 7 (283.2±7.5 g) whereas control animals maintained their normal weight trajectory during the 7-day period (from 330.5±7.0 g to 336.2±8.6 g). ANOVA revealed an effect of “group” [*F*_(1,10)_ = 12.7, *p*<0.01] accompanied by effect of “day” [*F*_(6,60)_ = 34.0, *p*<0.001] and “group×day” interaction [*F*_(6,60)_ = 35.3, *p*<0.001]. Following patch removal, the animals previously exposed to nicotine exhibited an abrupt weight increase at the two time points examined. According to ANOVA, a significant effect of “group” [*F*_(1,10)_ = 16.0, *p*<0.01] and “time” [*F*_(2,20)_ = 10.4, *p*<0.001] was accompanied to “group×time” interaction ([*F*_(2,20)_ = 4.4, *p*<0.05], Figure 1D).

### Experiment 2: Changes in tissue endocannabinoid levels during acute and protracted nicotine withdrawal

Exposure to intoxicating doses of nicotine elicits marked changes in AEA levels in various brain areas at the two time points measured (Table 1). Specifically, statistically detectable increases in AEA were observed in the amygdala [*F*_(3,23)_ = 4.4, *p*<0.05], hypothalamus [*F*_(3,23)_ = 3.4, *p*<0.05] and prefrontal cortex [*F*_(3,23)_ = 3.0, *p* = 0.05]. In the hippocampus AEA levels were decreased [*F*_(3,23)_ = 5.6, *p*<0.01], while no changes were observed in the caudate-putamen [*F*_(3,23)_ = 0.7, NS] and nucleus accumbens [*F*_(3,23)_ = 2.2, NS]. *Post hoc* analysis revealed changes of AEA levels mostly at 34 h, where they were significantly increased in the amygdala as well as hypothalamus (*p*<0.05), and a tendency to increase was observed in the prefrontal cortex (*p* = 0.09). In the hippocampus AEA was decreased only at 16 h (*p*<0.05).

**Table 1:** Levels of the endocannabinoids anandamide (AEA, pmol/g) in different brain structures during acute (16 h) and protracted (34 h) nicotine withdrawal phases.

AEA (pmol/g)	16 h		34 h	
	Control	Nicotine	Control	Nicotine
PFC	1.69±0.2	1.58±0.3	1.44±0.2	2.38±0.3
NAC	2.51±0.5	2.04±0.4	4.09±1.3	2.54±0.4
Amy	1.59±0.2	2.27±0.3	1.62±0.2	2.62±0.3*
CPU	1.94±0.3	2.08±0.1	1.75±0.1	2.14±0.2
Hippo	4.47±0.6	3.06±0.3*	3.44±0.5^#^	2.55±0.2
Hypo	4.13±0.4	4.73±1.4	4.69±0.8	8.39±1.3*

Results showed that major changes occurred for AEA particularly in the amygdala, the hypothalamus and the hippocampus. Values represent the mean (±SEM) of subjects previously treated with nicotine compared to controls at respective time points: *p<0.05.^#^ *p*<0.05, ^##^ *p*<0.01, difference from control group at 16 h time point; ^+^ *p*<0.05, difference from nicotine group at 16 h time point.

Variation in 2-AG levels were much less pronounced (Table 2). Overall ANOVA revealed a significant alteration in 2-AG values in the hippocampus [*F*_(3,23)_ = 6.9, *p*<0.01]. However, such difference was due to changes in 2-AG levels at 34 h compared to 16 h and was not the result of nicotine intoxication. A trend toward 2-AG increase in the amydgala [*F*_(3,23)_ = 2.6, *p* = 0.08] was also noted. No differences were detected in the hypothalamus [*F*_(3,23)_ = 1.0, NS]; nucleus accumbens [*F*_(3,23)_ = 0.4, NS]; prefrontal cortex [*F*_(3,23)_ = 0.2, NS] and caudate-putamen [*F*_(3,23)_ = 1.5, NS].

**Table 2:** Levels of 2-arachydonyl-*sn*-glycerole (2-AG, pmol/mg).

2-AG (pmol/mg)	16 h		34 h	
	Control	Nicotine	Control	Nicotine
PFC	2.87±0.4	2.95±0.4	2.59±0.5	3.03±0.3
NAC	0.82±0.2	0.89±0.2	1.10±0.4	0.82±0.1
Amy	2.88±0.4	3.89±0.4	2.30±0.7	4.32±0.7
CPU	1.02±0.1	1.18±0.1	1.35±0.1	1.20±0.1
Hippo	2.75±0.4	2.45±0.2	1.41±0.2^##^	1.62±0.2^+^
Hypo	5.57±1.0	6.54±0.7	4.64±1.6	6.57±0.7

For detailed statistics, see Results. PFC, prefrontal cortex; NAC, nucleus accumbens; Amy, amygdala; CPU, caudate putamen; Hippo, hippocampus; Hypo, hypothalamus.

### Experiment 3: URB597 did not alter somatic withdrawal signs of nicotine abstinence or locomotor activity and withdrawal-induced weight gain

**Figure 2:**
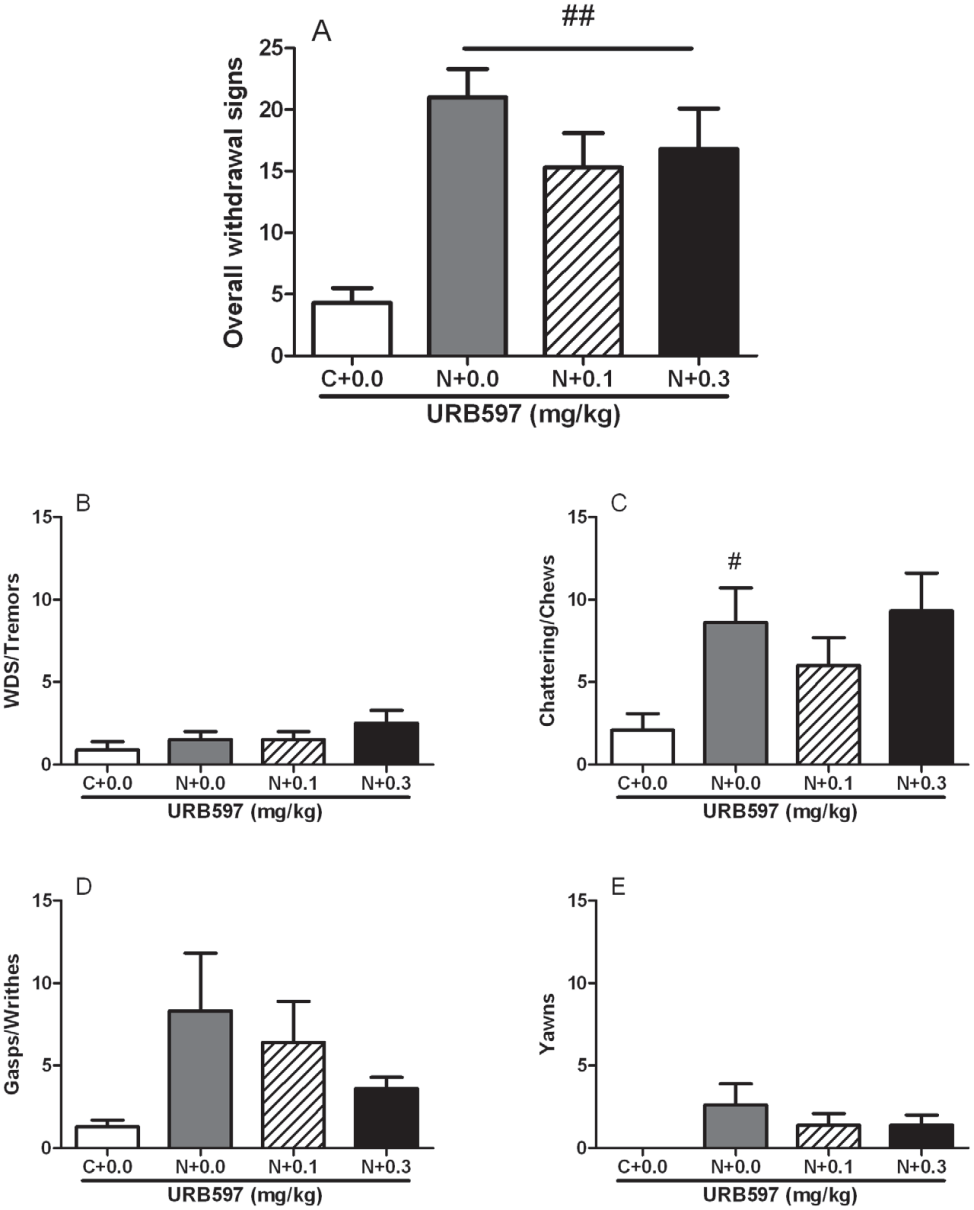
Spontaneous nicotine withdrawal measured 16 hours after nicotine patches (5.2 mg/rat/day) removal was not prevented by administration of URB597 (0.0, 0.1 and 0.3 mg/kg, i.p.). (**A**) overall withdrawal symptoms, (**B**) wet dog shakes/tremors, (**C**) chattering/chews, (**D**) gasp/writhes, (**E**) yawns. Values represent the mean (±SEM) of N = 8 subjects per group. #*p*<0.05, ##*p*<0.01, difference vs. “C+0.0” group.

Overall ANOVA revealed that 7-day exposure to transdermal nicotine patches induced a significant ([*F*_(3,28)_ = 8.0, *p*<0.01]) exacerbation of spontaneous withdrawal after 16 h from nicotine patch removal. As shown by *post hoc* tests, treatment with URB597 failed to decrease total abstinence score (Figure 2A).

Analysis of individual somatic signs showed a significant overall increase in the category chatter/chews [*F*_(3,28)_ = 3.0, *p*<0.05]. Post hoc analysis revealed a significant difference (*p*<0.05, Figure 2C) between naïve nicotine controls and rats exposed to nicotine and treated with URB597 vehicle. The occurrence of wet dog shakes/tremors ([*F*_(3,28)_ = 1.2, NS], Figure 2A), gasp/writhes ([*F*_(3,28)_ = 2.0, NS], Figure 2D) and yawns ([F_(3,28)_ = 1.8, NS], Figure 2E) was statistically unaffected and no difference was detected between the different groups. A clear trend to increase of these signs following nicotine cessation was always observed. A weak tendency of URB597 in reducing gasp/writhes and yawns in nicotine-exposed rats was noticed.

Locomotor activity in the open field was also analyzed (Supplemental Table S4). Analysis of variance revealed a non-significant overall difference for all the examined variables: distance travelled [*F*_(3,28)_ = 1.5, NS], immobility time [*F*_(3,28)_ = 2.0, NS], number of rearings [*F*_(3,28)_ = 0.4, NS]. According to results of Experiment 1, a trend to a drop in locomotion was observed in nicotine dependent animals compared to controls. URB597 showed a slight non-significant trend to reverse the locomotor suppressive effects associated to acute nicotine abstinence.

Body weight data at the time of patch removal (T = 0) and 16 h during withdrawal (T = 16) are shown in Supplemental Table S5. ANOVA revealed a main effect of “group” [*F*_(3,28)_ = 14.1, *p*<0.001] as well as a main effect of “time” [*F*_(1,28)_ = 72.5, *p*<0.001], and “group×time” interaction [*F*_(3,28)_ = 3.1, *p*<0.05]. On *post hoc* analysis, all three nicotine treated groups displayed body weight gain regardless of URB597 treatment (*p*<0.001).

### Experiment 4: URB597 reversed withdrawal-induced anxiety in the EPM test

**Figure 3:**
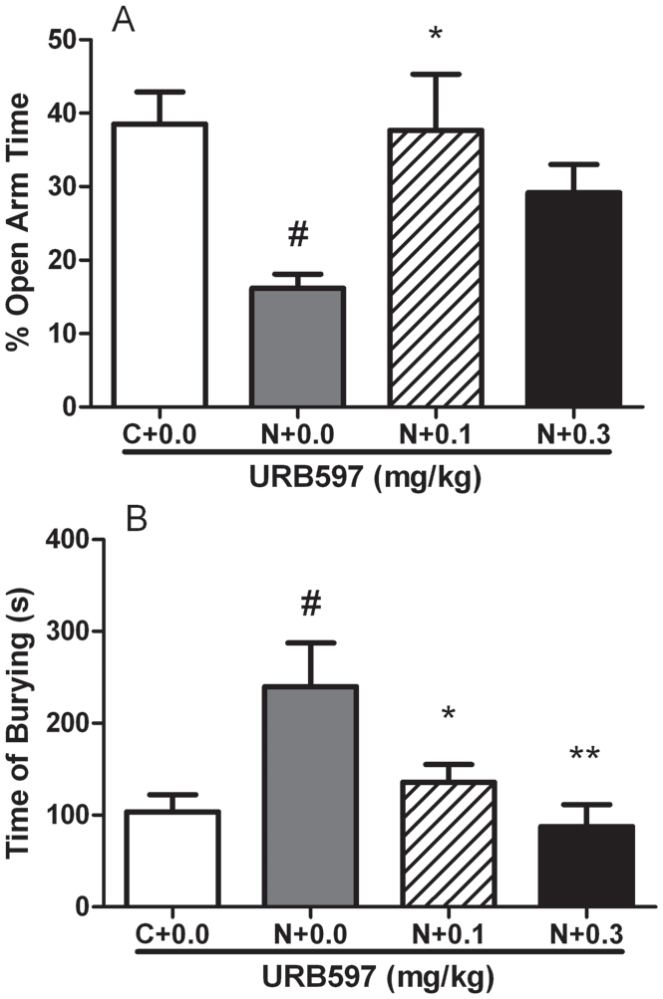
URB597 blocked nicotine withdrawal-induced anxiety-like behavior, as assessed in the elevated plus-maze and in the shock-probe defensive burying tests 34 hours after nicotine discontinuation. (**A**) Animals previously exposed to nicotine (N+0.0) showed significant reduction in percent (%) open arm time compared to controls (C+0.0). URB597 returned % open arms time to control levels. Values represent the mean (±SEM) of N = 10–11 subjects per group. (**B**) Animals previously exposed to nicotine (N+0.0) showed significant increase in burying time (seconds) compared to controls (C+0.0). URB597 dose-dependently returned burying time to control levels. Values represent the mean (±SEM) of N = 7–8 subjects per group; *p<0.05, **p<0.01 compared to nicotine exposed receiving vehicle; #*p*<0.05, difference from non-nicotine exposed controls.

In agreement with Experiment 1, a potent anxiogenic-like effect of nicotine withdrawal was detected at 34 h from patch removal, which was reversed by URB597. ANOVA revealed a significant overall effect in the percent of time spent in the open arms [*F*_(3,39)_ = 4.4, *p*<0.01]. *Post hoc* analysis demonstrated that URB597 significantly reversed the effect of nicotine withdrawal at 0.1 mg/kg (*p*<0.05) and a clear trend was observed at 0.3 mg/kg (*p* = 0.07, Figure 3A). The number of entries onto the open arms of the maze was not affected [*F*_(3.39)_ = 0.4, NS]. Closed-arm entries, a measure of locomotor activity, did not differ between control and URB597-treated animals ([*F*_(3,39)_ = 1.5, NS], Table S6).

Additional nicotine naïve rats were used to test URB597 in the EPM under basal conditions. Results evidenced that at doses that reduced nicotine withdrawal-induced anxiety (0.1, 0.3 mg/kg), URB597 did not affect elevated plus maze performance in nicotine naïve animals. ANOVA revealed lack of significance for all the dependent variables examined: percent of time spent in the open arms [*F*_(2,24)_ = 0.3, NS]; percent of open arm entries [*F*_(2,24)_ = 0.3, NS]; closed arm entries [*F*_(2,24)_ = 0.8, NS], Supplemental Table S7.

### Experiment 5: URB597 prevented withdrawal-induced enhancement of defensive burying

In the shock-probe defensive burying paradigm neither latency to approach the probe for the first time nor the number of times rats receiving shock from contacting the probe was modified by nicotine abstinence, and as shown in Supplemental Table S8 ANOVA revealed non-significant group differences ([*F*_(3,27)_ = 0.2, NS] and ([*F*_(3,27)_ = 0.7, NS], respectively). More importantly, ANOVA revealed an overall difference in the time spent burying ([*F*_(3,27)_ = 5.4, *p*<0.01]). *Post hoc* analysis showed an increase in burying time of nicotine-exposed rats receiving vehicle compared to nicotine naïve animals (*p*<0.01). In animals exposed to nicotine but pre-treated with URB597 0.1 (*p*<0.05) and 0.3 mg/kg (*p*<0.01), a significant reversal nicotine withdrawal-induced burying was observed (Figure 3B).

## Discussion

We found that a seven-day exposure to chronically implanted transdermal nicotine patches produces sufficiently elevated levels of blood nicotine and cotinine leading to a nicotine dependent state characterized by spontaneous formation of a severe withdrawal syndrome which includes somatic and affective components. The present findings confirm previous studies in adolescent rats where long-lasting effects on locomotor activity and anxiety-like behaviors were described following chronic nicotine patch delivery [13], [45]. In addition, consistent with already published work [11], [12], [46], we found that the peak in the manifestation of somatic signs occurred 16 h after nicotine discontinuation. Among categories of observed somatic withdrawal signs, results showed statistically greater occurrence of teeth chattering/chews, gasp/writhes and wet dog shakes/tremors. Withdrawal was accompanied also by a drop in locomotor activity and a rapid body weight gain following nicotine discontinuation. These measures further reflect nicotine dependence severity. In humans, somatic manifestations of nicotine withdrawal are often accompanied by depressed mood, aversive motivational state and increased anxiety, whereas in laboratory animals withdrawal is associated to elevations in brain reward threshold suggesting diminished interest or pleasure in obtaining rewarding stimuli [5], [19], a symptom classically associated to depressed mood in humans. Previous studies in rats also showed heightened anxiety in the auditory startle reflex and social interaction tests in rats made dependent to nicotine [47], [48], [49]. Consistent with these observation at 34 h following the removal of nicotine patches, a time point that corresponds to the protracted withdrawal phase, we observed significant increase in anxiety-like symptoms both in the EPM and in the defensive burying test.

Evidence suggests that nicotine addiction is linked to a modulation of the endocannabinoid signaling system in the brain. It has been shown, for example, that chronic exposure to nicotine leads to a decrease in AEA and 2-AG levels in midbrain, hippocampus, striatum and cerebral cortex, whereas increased AEA was detected in the limbic forebrain and brainstem [50]. Consistent with a role of the endocannabinoid system in nicotine addiction recent studies also showed that inhibition of FAAH, the major AEA degrading enzyme in the brain, interferes with nicotine discriminative properties, blunts nicotine reward, prevents its self-administration and reduces relapse [51], [52], [53]. Although this important evidence implicates endocannabinoid mechanisms in nicotine addiction, to our knowledge no data are currently available on the role of such mechanisms in nicotine withdrawal.

To test the hypothesis that nicotine abstinence is associated with alterations in endocannabinoid levels, in the present work the contents of AEA and 2-AG were examined in brain structures that are known to play a role in the regulation of negative reinforcement and drug withdrawal symptoms and that express elevated endocannabinoid activity as well as nicotinic acetylcholine receptors [22], [32], [54]. Such evaluation was carried out at two time points to evaluate endocannabinoid fluctuations associated with acute (16 h) and protracted (34 h) abstinence. The results show an increase in AEA levels in the amygdala, hypothalamus and prefrontal cortex, while reductions in AEA were detected in the hippocampus. Importantly, most changes in AEA levels occurred 34 h after nicotine exposure suggesting that anandamide alterations is likely associated to protracted abstinence. Contrary to AEA, 2-AG levels remained generally unaltered and a non-significant trend to an increase was only observed in the amygdala. In several studies including ours, it has been shown that somatic manifestations of nicotine withdrawal peak at about 16 h from nicotine exposure [11], [12], [46]. While, as observed here, at later time points (i.e., 34 h) affective withdrawal signs such as anxiety are predominant. Given that fluctuations in AEA levels have been detected primarily at 34 h we hypothesize that this endocannabinoid modulates affective rather than somatic components of withdrawal.

To explore the functional significance of AEA fluctuations in nicotine withdrawal, we tested the effect of URB597, a potent FAAH inhibitor, on both the somatic and the affective components of withdrawal. The results show that URB597 does not modify the expression of somatic withdrawal signs neither it affects withdrawal-induced hypolocomotion or body weight gain. While this finding confirms our hypothesis that AEA is not involved in the expression of body symptoms of nicotine withdrawal, on the other hand, it does not match with a previous study which examined the effects of Δ9-tetrahydrocannabinol (Δ^9^-THC ) on the incidence of nicotine withdrawal signs precipitated by mecamylamine or naloxone in mice [15]. These authors showed a clear protective effect of Δ^9^-THC in reducing global score of somatic signs. However, in our study we evaluated spontaneously occurring acute withdrawal compared to these previous studies where a more severe antagonist-precipitated abstinence was studied [12], [19]. Discrepancy may be also due to possible differences in pharmacological properties of direct agonists at CB1 receptors compared to agents (i.e., FAAH inhibitors) which activate these receptors following indirect pathways. Indeed, it was shown that a systemic dose of 0.3 mg/kg that maximally blocks FAAH, failed to mimic exogenous anandamide in producing catalepsy, hypothermia and hyperphagia [34]. Moreover, the same study by Balerio *et al* showed that the CB1 receptor antagonist rimonabant failed to precipitate somatic signs in nicotine dependent mice and, in a recent study that used FAAH KO mice [16], somatic signs were 2-fold higher compared with wild-type controls whereas the pharmacological inhibition of FAAH by URB 597 produced no effect at moderate doses, thus supporting our data. Altogether these findings suggest a complex role of endogenous cannabinoid system in the physiological control of the somatic manifestations of nicotine withdrawal but clearly point to lack of protective properties by FAAH inhibitors .

The relevance of endocannabinoid mechanisms in the regulation of affective signs of nicotine withdrawal was instead confirmed by data showing that at 34 h treatment with URB597 prevented the anxiogenic-like response associated with nicotine discontinuation. Two different behavioral paradigms were used to assess anxiety-like behavior: the EPM and the shock-probe defensive burying tests. While the former is thought to reflect generalized anxiety disorders [39], the latter commonly measures fear-like responses generated by exposure to stressful environmental conditions [40]. In these tests, anxiety associated to nicotine withdrawal was revealed by a decreased percent of time spent exploring the open arms of the EPM and by an increase in time spent burying, respectively. URB597 treatment significantly enhanced open arms exploration at the dose of 0.1 mg/kg while markedly reduced the burying time in a dose-dependent manner. These findings indicate that the anxiolytic effect of URB597 is specific and does not depend upon drug-induced alterations in locomotor behavior. In fact, if in the EPM generalized hypolocomotion may reduce exploration thus mimicking an anxiogenic-like condition, in the defensive burying test hypolocomotion would result in burying reduction that is a measure of anxiolytic effects.

Our results are consistent with evidence that direct activation of cannabinoid receptors by Δ^9^-THC attenuates anxiogenic- but not anxiolytic-like responses following acute nicotine administration. [31]. Moreover, the findings extend previous observations indicating that URB597 has particularly pronounced anxiolytic-like effect if anxiety is associated with aversive conditions such as stressful environments or drug withdrawal [36], [55]. However, in earlier reports anxiolytic-like effects of URB597 under basal conditions were also described in mice [34], [56] and rats [53], [57]. Notably, in the EPM in rats this effect was found at doses higher (1.0 mg/kg) than those found to be effective in withdrawing rats (0.1 mg/kg) in our study [57]. Based on these findings it may be argued that illumination of the testing arena, habituation of the animals to the testing environment and their handling may determine the anxiolytic efficacy of URB597. It is plausible, in fact, that FAAH inhibition may result in anxiolytic action only when given to animals exposed to highly stressful stimuli.

AEA is a local neuromodulator whose release is thought to be regulated “on demand” by the activation of a variety of neuronal substrates [54]. Upon its release, AEA is primarily degraded by intracellular FAAH activity. Hence, blockade of this enzyme by URB597 results in selective augmentation of AEA-mediated transmission in those neural circuits in which this endocannabinoid is recruited. In the present study, we found that protracted nicotine withdrawal is associated with increased anxiety and augmented AEA levels. On the other hand, inhibition of FAAH by URB597, leading to further increase in AEA mediated neurotransmission results in a reversal of anxiety-like responses associated to nicotine withdrawal. These findings can be reconciled based on the hypothesis that during withdrawal AEA is released to restore physiological mechanisms temporarily disrupted by the abrupt cessation of nicotine administration. However, if this increase in AEA-mediated activity is not sufficient to counteract the functional imbalance caused by sudden nicotine discontinuation it is only after administration of URB597, and subsequent accumulation of AEA, that is possible to observe an effect on nicotine withdrawal. Consistent with the anxiolytic role of endocannabinoids, current literature suggests that central administration of AEA, similar to URB597 results in marked anxiolytic-like effects [58]. Similarly, the AEA transporter inhibitor AM404 was clearly shown to blunt mice escape reactions associated with spontaneous opiate withdrawal [59].

Exogenous stimulation of nicotinic acetylcholine receptors or lack of their activation, may be critical for fluctuations of endocannabinoid contents in the brain. The finding that alpha 7 nicotinic receptors initiate AEA formation in cortical neurons strengthens this view [60]. In the present study, we found that the amygdala is one of the areas where changes in endocannabinoid level were most remarkable, and where AEA, and to a lesser extent also 2-AG, showed an increase following nicotine discontinuation. Given that the amygdala is involved in the regulation of negative affect and emotions [61], this finding may indicate a role for AEA in this area in the modulation of anxiety associated to nicotine withdrawal.

Nicotine withdrawal is associated to a region-specific enhancement of neuronal activity in the central nucleus of the amygdala [62], and is accompanied by a selectively increase in adenylyl cyclase activity in this brain area [63]. Since activation of CB_1_ receptors causes adenylyl cyclase inhibition, it may be speculated that increased AEA content in the amygdala serves to counterbalance withdrawal-induced stimulation of this intracellular signaling pathway. A role of corticotrophin releasing hormone (CRH), known to mediate endocrine, physiological and behavioral responses to stress [64], is also possible. CRH is thought to play a central role of the modulation of withdrawal from a variety of drugs including alcohol [65], cocaine [66], cannabis [67] and nicotine [68], [69]. On the other hand, the endocannabinoid system has been suggested to act as an anti-stress system [70], [71]. Hence, its activity during withdrawal may be increased in the attempt to counteract for heightened CRH neurotransmission. During nicotine withdrawal a significant increase in AEA levels were also detected in the hypothalamus. Considering that elevated contents in hypothalamic AEA may take part to the regulation of neuroendocrine response to stress [70], it may be possible that dysregulation of the endocannabinoid system in this area may take part to alterations of hypothalamic-pituitary-adrenal axis activity associated to nicotine withdrawal [72]. Finally, the finding that AEA levels are decreased in hippocampus may be indicative of an imbalance between the endocannabinoid and the cholinergic system in this area. Notably, an equilibrium between cholinergic and endocannabinoid neurotransmission within the hippocampus seems to be crucial for the homeostasis of emotional behavior and, in turn, a misbalance, might result in a variety of emotional and mood-related disorders [73].

In conclusion, we show here that a seven-day exposure to chronically implanted transdermal nicotine patches induces nicotine dependence characterized by spontaneous formation of a severe withdrawal syndrome, which includes somatic and affective components. Withdrawal was associated to marked changes in AEA levels in several brain areas, while 2-AG content was only modestly affected. Previous studies demonstrated that FAAH inhibition results in marked reduction in nicotine self-administration and relapse to drug seeking [50], [51], [52], while here we demonstrated that the pharmacological inhibition of FAAH by URB597 decreased anxiety associated to protracted nicotine withdrawal. Together, these findings point to the possibility that FAAH inhibition may represent an effective pharmacological strategy for the treatment of nicotine addiction. Considering that negative affective states associated with nicotine withdrawal is a major concern for smoking cessation, this approach could result particularly promising.

## Supporting Information

Table S1 Somatic withdrawal signs observed 16 hours after chronic nicotine discontinuation. Overall withdrawal signs as well as single categories of the examined physical symptoms (teeth-chattering/chews, gasps/writhes, shakes/tremors) were increased in animals previously exposed to nicotine. Difference from controls: *p<0.05, **p<0.01.
(DOC)

Table S2 Open field performance at 16 hours from nicotine discontinuation. Nicotine exposed rats showed a decrease in locomotor activity and an increase in immobility time. Difference from controls: *p<0.05.
(DOC)

Table S3 Anxiety-like behavior measured by elevated plus maze at 34 hours from nicotine discontinuation. Percent (%) open arms was significantly decreased in nicotine exposed animals compared to controls. Difference from controls: *p<0.05.
(DOC)

Table S4 Open field performance in nicotine exposed rats treated with URB597 vehicle (N+0.0) or with 0.1 (N+0.1) and 0.3 mg/kg (N+0.3) of URB597. Difference from controls (C+0.0) was not significant.
(DOC)

Table S5 Rats previously exposed to nicotine showed increased weight gain compared with controls at 16 hours (T = 16 h) from patches removal (T = 0 h). Nicotine naïve control (C+0.0), animals exposed to nicotine and treated with URB597 vehicle (N+0.0) or with 0.1 (N+0.1) and 0.3 mg/kg (N+0.3) of URB597. Difference from Controls: ***p<0.001.
(DOC)

Table S6 Anxiety-related (percent open arm time and entries) and locomotor-related (closed arms entries) variables on EPM performance at 36 hours from nicotine discontinuation. Nicotine naïve control (C+0.0), animals exposed to nicotine and treated with URB597 vehicle (N+0.0) or with 0.1 (N+0.1) and 0.3 mg/kg (N+0.3) of URB597. A non significant trend to reduction in % open arm entries was observed in rats treated with nicotine and URB597. *p<0.05, compared to nicotine exposed receiving vehicle; #*p*<0.05, difference from non-nicotine exposed controls.
(DOC)

Table S7 EPM performance following URB597 treatment (0.0, 0.1, 0.3 mg/kg) in control non-withdrawing animals.
(DOC)

Table S8 Defensive burying performance scored 36 hours from nicotine discontinuation. The number of probe approaches and latency to bury was not different between groups. Nicotine naïve control (C+0.0), animals exposed to nicotine and treated with URB597 vehicle (N+0.0) or with 0.1 (N+0.1) and 0.3 mg/kg (N+0.3) of URB597.
(DOC)
