# Supplementary material for: Endocannabinoid Regulation of Acute and Protracted Nicotine Withdrawal: Effect of FAAH Inhibition
Source: PLoS One. 2011 Nov 30;6(11):e28142. doi: 10.1371/journal.pone.0028142 (PMC3227620; doi:10.1371/journal.pone.0028142)
Supplement: Table S1 — Somatic withdrawal signs observed 16 hours after chronic nicotine discontinuation. Overall withdrawal signs as well as single categories of the examined physical symptoms (teeth-chattering/chews, gasps/writhes, shakes/tremors) were increased in animals previously exposed to nicotine. Difference from controls: *p<0.05, **p<0.01. (DOC) [file pone.0028142.s001.doc]

**Table S1**

| *Category* | Control | Nicotine exposed |
| --- | --- | --- |
| Teeth-chattering/chews | 2.0±0.9 | 6.0±1.5* |
| Gasps/Writhes | 1.8±0.5 | 6.5±1.1** |
| Shakes/Tremors | 0.5±0.2 | 2.3±0.3** |
| Overall | 4.3±1.3 | 14.8±2.0** |
