# Supplementary material for: Endocannabinoid Regulation of Acute and Protracted Nicotine Withdrawal: Effect of FAAH Inhibition
Source: PLoS One. 2011 Nov 30;6(11):e28142. doi: 10.1371/journal.pone.0028142 (PMC3227620; doi:10.1371/journal.pone.0028142)
Supplement: Table S2 — Open field performance at 16 hours from nicotine discontinuation. Nicotine exposed rats showed a decrease in locomotor activity and an increase in immobility time. Difference from controls: *p<0.05. (DOC) [file pone.0028142.s002.doc]

**Table S2**

| *Locomotor Activity* | Control | Nicotine exposed |
| --- | --- | --- |
| Total distance traveled (cm) | 3475.8±262.3 | 2538.8±344.1* |
| Immobility time (s) | 342.3±14.1 | 397.2±17.2* |
| Rearings (s) | 134.3±10.7 | 126.3±19.5 |
