# Supplementary material for: Endocannabinoid Regulation of Acute and Protracted Nicotine Withdrawal: Effect of FAAH Inhibition
Source: PLoS One. 2011 Nov 30;6(11):e28142. doi: 10.1371/journal.pone.0028142 (PMC3227620; doi:10.1371/journal.pone.0028142)
Supplement: Table S3 — Anxiety-like behavior measured by elevated plus maze at 34 hours from nicotine discontinuation. Percent (%) open arms was significantly decreased in nicotine exposed animals compared to controls. Difference from controls: *p<0.05. (DOC) [file pone.0028142.s003.doc]

**Table S3**

| *Withdrawal-induced anxiety* | Control | Nicotine exposed |
| --- | --- | --- |
| Open arm time (%) | 39.2±5.7 | 19.4±5.2* |
| Open arm entries (%) | 46.3±0.6 | 29.2±7.9 |
| Closed arm entries | 12.0±0.9 | 10.3±2.4 |
