# Supplementary material for: Endocannabinoid Regulation of Acute and Protracted Nicotine Withdrawal: Effect of FAAH Inhibition
Source: PLoS One. 2011 Nov 30;6(11):e28142. doi: 10.1371/journal.pone.0028142 (PMC3227620; doi:10.1371/journal.pone.0028142)
Supplement: Table S4 — Open field performance in nicotine exposed rats treated with URB597 vehicle (N+0.0) or with 0.1 (N+0.1) and 0.3 mg/kg (N+0.3) of URB597. Difference from controls (C+0.0) was not significant. (DOC) [file pone.0028142.s004.doc]

**Table S4**

| *Locomotor activity* | C+0.0 | N+0.0 | N+0.1 | N+0.3 |
| --- | --- | --- | --- | --- |
| Distance (cm) | 3516.5±258.9 | 2655.9±247.5 | 2803.5±317.2 | 3254.8±428.3 |
| Immobility (s) | 349.0±12.9 | 401.9±14.7 | 397.0±19.4 | 381.1±19.8 |
| Rearings (s) | 133.3±8.3 | 111.6±7.8 | 128.4±23.7 | 123.5±15.1 |
