# Supplementary material for: Endocannabinoid Regulation of Acute and Protracted Nicotine Withdrawal: Effect of FAAH Inhibition
Source: PLoS One. 2011 Nov 30;6(11):e28142. doi: 10.1371/journal.pone.0028142 (PMC3227620; doi:10.1371/journal.pone.0028142)
Supplement: Table S5 — Rats previously exposed to nicotine showed increased weight gain compared with controls at 16 hours (T = 16 h) from patches removal (T = 0 h). Nicotine naïve control (C+0.0), animals exposed to nicotine and treated with URB597 vehicle (N+0.0) or with 0.1 (N+0.1) and 0.3 mg/kg (N+0.3) of URB597. Difference from Controls: ***p<0.001. (DOC) [file pone.0028142.s005.doc]

**Table S5**

| *Weight (g)* | C+0.0 | N+0.0 | N+0.1 | N+0.3 |
| --- | --- | --- | --- | --- |
| T=0 h | 343.4±12.1 | 270.1±7.6 | 282.6±10.1 | 270.5±3.7 |
| T=16 h | 347.4±11.7 | 283.5±8.8*** | 293.0±9.1*** | 281.4±3.4*** |
