# Supplementary material for: Endocannabinoid Regulation of Acute and Protracted Nicotine Withdrawal: Effect of FAAH Inhibition
Source: PLoS One. 2011 Nov 30;6(11):e28142. doi: 10.1371/journal.pone.0028142 (PMC3227620; doi:10.1371/journal.pone.0028142)
Supplement: Table S6 — Anxiety-related (percent open arm time and entries) and locomotor-related (closed arms entries) variables on EPM performance at 36 hours from nicotine discontinuation. Nicotine naïve control (C+0.0), animals exposed to nicotine and treated with URB597 vehicle (N+0.0) or with 0.1 (N+0.1) and 0.3 mg/kg (N+0.3) of URB597. A non significant trend to reduction in % open arm entries was observed in rats treated with nicotine and URB597. *p<0.05, compared to nicotine exposed receiving vehicle; #p<0.05, difference from non-nicotine exposed controls. (DOC) [file pone.0028142.s006.doc]

**Table S6**

| *EPM variables* | C+0.0 | N+0.0 | N+0.1 | N+0.3 |
| --- | --- | --- | --- | --- |
| Open arm time (%) | 38.5±4.4 | 16.2±1.9# | 37.7±7.6* | 29.2±3.8 |
| Open arm entries (%) | 48.1±0.4 | 30.8±3.6 | 46.8±1.6 | 47.4±0.5 |
| Closed arm entries | 12.1±1.0 | 9.9±1.2 | 8.5±1.5 | 9.9±0.9 |
