# Supplementary material for: Endocannabinoid Regulation of Acute and Protracted Nicotine Withdrawal: Effect of FAAH Inhibition
Source: PLoS One. 2011 Nov 30;6(11):e28142. doi: 10.1371/journal.pone.0028142 (PMC3227620; doi:10.1371/journal.pone.0028142)
Supplement: Table S7 — EPM performance following URB597 treatment (0.0, 0.1, 0.3 mg/kg) in control non-withdrawing animals. (DOC) [file pone.0028142.s007.doc]

**Table S7**

| *EPM variables* | 0.0 | 0.1 | 0.3 |
| --- | --- | --- | --- |
| Open arm time (%) | 14.9±4.1 | 16.4±4.9 | 12.1±3.2 |
| Open arm entries (%) | 21.4±6.2 | 24.3±4.8 | 18.6±3.7 |
| Closed arm entries | 11.2±1.0 | 11.0±0.5 | 12.7±1.4 |
