# Supplementary material for: Endocannabinoid Regulation of Acute and Protracted Nicotine Withdrawal: Effect of FAAH Inhibition
Source: PLoS One. 2011 Nov 30;6(11):e28142. doi: 10.1371/journal.pone.0028142 (PMC3227620; doi:10.1371/journal.pone.0028142)
Supplement: Table S8 — Defensive burying performance scored 36 hours from nicotine discontinuation. The number of probe approaches and latency to bury was not different between groups. Nicotine naïve control (C+0.0), animals exposed to nicotine and treated with URB597 vehicle (N+0.0) or with 0.1 (N+0.1) and 0.3 mg/kg (N+0.3) of URB597. (DOC) [file pone.0028142.s008.doc]

**Table S8**

| *Defensive burying* | C+0.0 | N+0.0 | N+0.1 | N+0.3 |
| --- | --- | --- | --- | --- |
| Latency (s) | 123.4±15.3 | 108.4±28.8 | 109.3±15.8 | 129.3±23.0 |
| Probe approaches | 2.6±0.4 | 3.4±0.4 | 2.6±0.3 | 2.9±0.5 |
